# Isolated medial patellofemoral ligament reconstruction improves static bipedal balance control in young patients with recurrent lateral patellar instability

**DOI:** 10.1186/s13018-023-04272-9

**Published:** 2023-10-12

**Authors:** Fenghua Tao, Hai Tao, Lin Jin, Haijun Gao, Yue Luo, Zheng Zhang

**Affiliations:** 1 Department of Orthopedics, Renmin Hospital of Wuhan University, 238, Jiefang Road, Wuchang District, Wuhan, 430060 Hubei China; 2 Department of Emergency, Renmin Hospital of Wuhan University, Wuhan, China

**Keywords:** Medial patellofemoral ligament, Recurrent patellar instability, Balance control, Posturography

## Abstract

**Background:**

Knee stability can be safely and reliably restored using medial patellofemoral ligament (MPFL) reconstruction, which is widely recognized in patients with recurrent lateral patellar instability. However, the literature regarding its influence on static balance control is limited. Thus, this study aimed to assess the impact of MPFL reconstruction on balance control and determine its functional significance.

**Methods:**

The study comprised 26 patients with recurrent lateral patellar instability, scheduled for MPFL reconstruction, and 26 matched healthy controls who underwent double-leg stance static posturographic tests pre- and postoperatively on a vertical force platform. Four test conditions were performed with their eyes open and closed, without and with foam support to evaluate the balance control of all participants. The International Knee Documentation Committee subjective knee form, Lysholm knee scoring scale, Tampa scale for kinesiophobia, and active range of motion of the affected knee were synchronously obtained and assessed.

**Results:**

More postural sway was observed in patients compared to the healthy controls, 11 ± 5 days preoperatively (*p* < 0.01). However, 374 ± 23 days postoperatively, postural sway between the patients and control subjects was comparable (*p* > 0.05). Patients following MPFL reconstruction demonstrated better postural stability (*p* < 0.01). Significant ameliorations were found in all clinical assessments in the study patients postoperatively (*p* < 0.01).

**Conclusions:**

Patients with recurrent lateral patellar instability have inefficient balance control. Static bipedal balance control can be improved under surface perturbation in these patients one year after isolated MPFL reconstruction that enhances the possibility of normal restoration of postural stability. Structural recovery of the ligament could help restore the sensorimotor efficiency and generate the compensatory and anticipatory balance regulation strategies, thereby improving joint function.

## Introduction

A common orthopedic complaint in the young population is lateral patellar instability, with an overall injury rate of 43 per 100,000 adolescent athletic patients [1, 2]. In general, patellar instability involves subluxation and dislocation. The standard of care for first-time dislocation is nonoperative treatment [3]. With an increased understanding of knee stability, conservative treatment of patellar instability is often found to be trivial as a 17% to 49% recurrence rate is observed in patients who sustain a primary or repeated patellar dislocation [4, 5]. When conservative therapy is unable to offer a satisfactory outcome, surgical treatment for recurrent patellar instability may be recommended [6, 7]. The appropriate surgical intervention is selected based on underlying structural anatomical abnormalities or insufficient soft tissue restraints [8]. To rectify significant anatomical and morphological abnormalities, procedures such as tibial tubercle osteotomy, rotational osteotomy, trochleoplasty, or patellar tendon shortening are commonly used individually or concomitantly [9–11]. For patients without anatomical abnormalities, the first choice for treating recurrent lateral patellar instability currently is a medial patellofemoral ligament (MPFL) reconstruction [12].

The MPFL is the primary medial soft tissue stabilizer preventing lateral displacement of the patella, as an essential structure contributing to maintaining patellar stability, providing up to 50% of the total restraint force from 0° to 30° of knee flexion, and accounting for approximately 60% of the stabilizing forces on the patellofemoral joint [13–15]. As a leading reference for evaluating the effect of treatment and rehabilitation and the level of return to sport, balance control in patients with sports injuries guides targeted therapies and exercises, assisting surgeons and physiotherapists in their decision-making [16]. Asaeda et al. detected that the gait kinematics in patients with recurrent patella dislocation having a preoperative deficit returns to normal, 1 year following MPFL reconstruction compared with controls [17]. In contrast, Shams et al. reported that patients undergoing MPFL reconstruction exhibit deficits in knee kinematics and kinetics for approximately 11 months postoperatively [18]. Although the importance of lower limb dynamic stability has been elucidated [19], less attention has been paid to the influence of MPFL reconstruction on static balance control in patients with recurrent patellar instability and the level of recovery of patients’ balance control after surgery. The lack of information regarding the static balance control after MPFL reconstruction prompts us to assess the influence of this type of procedure on patients’ postural stability to provide a reference in the management of disease treatment and rehabilitation.

This study aimed to compare the balance control performances before and after MPFL construction using the posturographic platform between the study patients and healthy controls. It was hypothesized that patients may have less efficient balance control performance compared to control subjects, preoperatively, which could be improved postoperatively. The study secondarily aimed to investigate the changes in balance control as well as the clinical manifestations of the affected knee before and after MPFL reconstruction in the study patients. It was hypothesized that patients may have better postural stability postoperatively owing to the recovery of ligament morphology and joint function.

## Materials and methods

### Research design

An observational case–control study design was conducted comparing the variations in balance control before and after MPFL reconstruction in patients with recurrent lateral patellar instability by performing posturography along with the normal scheduled surgical planning. The matched healthy controls were measured simultaneously to serve as a reference for the preoperative injury and postoperative recovery levels. After the patients completed the posturography at each testing phase, clinical assessments, including the International Knee Documentation Committee subjective knee form (IKDC) [20], Lysholm knee scoring scale (Lysholm) [21], Tampa scale for kinesiophobia (TSK) [22], and active range of motion (ROM) of the affected knee were performed. The IKDC and Lysholm scoring scale comprising ten and eight evaluation items, respectively, evaluate the symptoms, function, and sports activity of the affected knees using a questionnaire. Each item score is added up and converted into a total score ranging from 0 to 100, with 100 indicating the absence of symptoms with no daily life and sports activities limitations. The TSK rating scale comprises 17 self-reported items, designed to assess the reinjury fear owing to sports and daily activities. Each item provides a 4-point Likert scale. The item sum produces a total score from 0 to 51, with higher scores representing more fear. Active ROM indicates active knee flexion from full extension to the maximum tolerable angle by the patients.

The protocol and design of this observational study were reviewed and approved by the medical ethical committee of Renmin Hospital of Wuhan University. Each participant signed a written informed consent before the study commencement.

### Participants

The patients with recurrent lateral patellar instability reporting to the orthopedic clinic and emergency department of Renmin Hospital of Wuhan University were included. A single orthopedic surgeon assessed the patients based on their history of patellar instability, radiographs, computed tomography (CT) scans, and isolated MPFL reconstruction uniform standards [23–25]. The inclusion criteria were as follows: (1) patients with more than one patellar instability episode of dislocation and/or subluxation (displacement of more than 50% of the patellar width at 30° of knee flexion); (2) those without significant anatomical abnormalities; and (3) those with failure after 6 months of conservative treatment. Exclusion criteria were as follows: (1) trochlear angle > 145°; (2) Quadriceps angle (Q angle) > 17° in men or > 20° in women; (3) tibial tuberosity–trochlear groove distance > 20 mm; (4) Caton–Deschamps index > 1.2; (5) obvious J-tracking during knee extension; (6) femoral anteversion > 25° and/or external tibial torsion > 40°; (7) degenerative patellofemoral osteoarthritis of grades III or IV; (8) a history of patellar stabilization surgery on either knee; (9) bilateral patellar instability; and (10) other musculoskeletal disorders, dysopia, neurologic impairments, and severe depressive syndromes. Healthy controls with no lower extremity pathology and injury history were recruited from local high schools and sports academies using advertisements.

### MPFL reconstruction

MPFL reconstruction was performed by the same surgeons with a recommended surgical technique [24, 26]. An autologous semitendinosus tendon was used as a graft for the MPFL reconstruction. To avoid the patellar fracture risk, the original patellar attachment was reconstructed using two horizontally implanted suture anchors (Healix advance BR, DePuy Mitek, Raynham, USA) to fix the graft at the medial edge of the patella. Then, a guide pin was used for locating the femoral insertion point of the new ligament before drilling according to Schöttle’s method [27]. To allow the graft to pass through the soft tissue to reach Schöttle’s point, a subcutaneous tunnel was created. A full range of knee motion was performed to ensure isometry of the new ligament to avoid changes in graft length and tension. Finally, a bioabsorbable interference screw (Bio-INTRAFIX, DePuy Mitek, Raynham, USA) was used to finish the femoral fixation at 30° of knee flexion. To avoid the dual effects of rehabilitation and MPFL reconstruction on balance control, the patients did not engage in systematic physical therapy sessions after surgery. We only asked them to perform 20 min of free walking and 10 min of continuous passive motion at maximum tolerance of the affected knee three times a day for one week from the second postoperative day just to avoid bed rest complications such as swelling, stiffness, and muscle atrophy.

### Posturography

The static posturographic tests were performed by the same operator in a bright and quiet room in the hospital’s inpatient department before and after MPFL reconstruction to assess patients’ balance control capacity. Healthy controls were given the same tests as the study patients simultaneously. All participants were asked to stand bare feet on a vertical force platform (Win-Posturo, Medicapteurs, Balma, France) in a quiet upright position, keeping the body stable, with the feet abducted at 30°, heels 3 cm apart, and the arms along the body. Three strain-gauge force sensors were installed at the bottom of the platform to sense the participant’s body sway according to the displacement of the center of foot pressure (CoP) on a two-dimensional horizontal plane (recording time: 25.6 s, acquisition frequency: 40 Hz). The signal captured by the force sensors demonstrated the CoP trajectory, which was then quantitatively converted to digital form and recorded in the computer. A poorer balance control precision was indicated by a higher sway area (in mm^2^) covered by the CoP trajectory [28, 29]. The tests were conducted under four conditions (C1-C4) involving two visual (eyes open and closed) combined with two surface conditions (stable and foam support), which imitated different sensory input situations to evaluate the participant’s ability to use available sensory cues effectively and to suppress unavailable or disturbed sensory cues in balance control (Table 1). Participants were measured first on the firm platform with eyes open (C1) and closed (C2). They were then asked to stand on a 10 cm thick foam (70 kg/m^3^, Jinniu, JSC, Linyi, China) that was placed on the platform to simulate an environment where proprioception was disturbed, with their eyes open (C3) and closed (C4). Three trials were conducted in each test condition, and a mean value was recorded as the final result. To accurately assess participants’ adaptation and body balance regulation in response to internal and external constraints, a mean equilibrium score (MES) was introduced by summing the scores for each condition and then dividing the sum by four [29, 30].

**Table 1:** Posturographic test: determination of four testing conditions

Testing conditions	Unavailable or altered cues
C1: Eyes open, firm support	–
C2: Eyes closed, firm support	No vision
C3: Eyes open, foam support	Modified proprioception
C4: Eyes closed, foam support	No vision, modified proprioception

### Statistical analysis

The required sample size and power of study were calculated using the PASS software (NCSS, Kaysville, Utah, UAS). SPSS 22.0 software (IBM, Armonk, NY, USA) was used to analyze the research data. The Kolmogorov–Smirnov test was used to measure the normal distribution of quantitative data. The *χ*^2^ test was used to compare qualitative data that were expressed as numbers (*n*). The differences in quantitative demographic and anthropometric variables between study patients and healthy controls were compared using independent samples *t*-test. The patients’ clinical assessment results, including the IKDC, Lysholm, and TSK scores and the active ROM of the affected knee before and after MPFL reconstruction, were compared using the paired sample *t*-test. The differences in postural sway between patients and healthy controls during the same testing phase and the changes in postural sway of patients before and after surgery were compared using the linear mixed-effects model. The simple effect analyses were performed using post hoc comparisons by Bonferroni correction. The MES and clinical assessment results were correlated using regression analysis. Cohen’s d was used to calculate the effect sizes. The data were presented as mean ± standard deviation (SD) (normally distributed data). All statistical significance was specified as *p* < 0.05.

## Results

### Participants’ characteristics and measurement times

Based on the inclusion criteria, 26 patients and 26 controls were included in this study. The mean time from injury to surgery was 236 ± 57 days. Based on the measured mean and SD of the postural sway area, the sample comprising 52 participants could achieve 91.4% power of the study. Table 2 summarizes the demographic and anthropometric characteristics of participants. These parameters demonstrated no significant differences between patients and healthy controls (All *p* > 0.05). All participants were tested for balance control at a mean of 11 ± 5 and 374 ± 23 days before and after the patients underwent MPFL reconstruction, respectively.

**Table 2:** Demographic and anthropometric characteristics of participants (Mean ± SD)

Parameters	Study patients (*n* = 26)	Healthy controls (*n* = 26)	*p-*value
Sex (n), male/female	10/16	10/16	1.000
Age (years)	19.5 ± 2.7	20.9 ± 3.2	0.372
Height (cm)	165.6 ± 3.8	168.4 ± 5.1	0.625
Weight (kg)	59.7 ± 5.5	61.2 ± 8.2	0.214
BMI (kg/m^2^)	21.4 ± 1.6	22.1 ± 1.9	0.523
Q angle (degree)			
Male	14.7 ± 2.1	13.9 ± 3.0	0.243
Female	18.3 ± 1.5	16.9 ± 2.8	0.138
TT-TG distance (mm)	17.4 ± 1.8	14.4 ± 3.6	0.076
Trochlear angle (degree)	140.2 ± 3.4	135.7 ± 4.5	0.534
CDI	1.09 ± 0.08	1.07 ± 0.05	0.192
Femoral anteversion (degree)	12.4 ± 3.8	13.1 ± 2.7	0.285
External tibial torsion (degree)	27.6 ± 8.2	28.9 ± 9.4	0.376

BMI: body mass index; Q angle: quadriceps angle; TT-TG: tibial tuberosity–trochlear groove; CDI: Caton–Deschamps index

### Comparison of balance control between the study patients and healthy controls

**Fig. 1:**
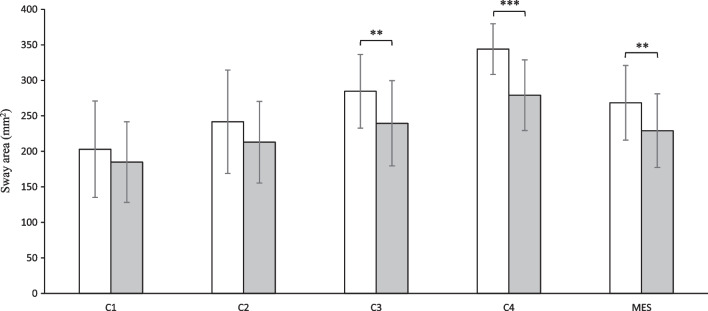
Mean values with standard deviations of postural sway area for four conditions (C1-C4) and mean equilibrium score (MES) in patients (white bars) and healthy controls (gray bars) before MPFL reconstruction; ^**^ *p* < 0.01, ^***^ *p* < 0.001

Table 3 shows the fixed effects, including time effect, group effect, and time-by-group interaction in the balance control test. Significant main effects were present for groups in C3, C4, and MES in the test (*p* < 0.05). The comparison of preoperative balance control between patients and control subjects is illustrated in Fig. 1. Patients and controls had undifferentiated balance control manifestations before MPFL reconstruction while standing on a stable support surface. In firm support and visually available condition (C1), patients had a postural sway area (203.1 ± 67.9 mm^2^) comparable to that of healthy controls (185.0 ± 57.0 mm^2^) (*p* = 0.304, Cohen’s d = 0.29). When visual dependence was lost (C2), patients still demonstrated a sway area (241.8 ± 72.8 mm^2^) not significantly different from controls (213.0 ± 57.5 mm^2^) (*p* = 0.119, Cohen’s d = 0.44). As the proprioceptive cues were modified, heterogeneities in balance control between patients and controls began to unfold. The study patients with their eyes open (C3), exhibited a larger sway area (284.6 ± 51.8 mm^2^) than that of controls (239.6 ± 60.1 mm^2^) (*p* = 0.004, Cohen’s *d* = 0.80). A more significant difference was observed between patients (344.1 ± 35.7 mm^2^) and controls (279.2 ± 49.7 mm^2^) when vision was completely absent in C4 (*p* < 0.001, Cohen’s *d* = 1.51). The patients had a worse balance control performance preoperatively (268.4 ± 52.5 mm^2^) in contrast with healthy controls (229.2 ± 51.9 mm^2^), as suggested by the overall comparison reflected by MES (*p* = 0.009, Cohen’s d = 0.75).

**Table 3:** Fixed effects under different testing conditions in balance control test

	Time effect		Group effect		Time × Group interaction	
	F	*p*	F	*p*	F	*p*
C1	0.295	0.590	0.520	0.474	2.464	0.123
C2	1.535	0.221	1.829	0.182	2.021	0.161
C3	0.141	0.709	6.248	0.016 ^*^	8.264	0.006 ^**^
C4	2.599	0.113	20.979	< 0.001 ^***^	5.077	0.029 ^*^
MES	1.913	0.173	4.942	0.031 ^*^	7.973	0.007 ^**^

C1-C4: four testing conditions in balance control test; MES: mean equilibrium score

^*^p < 0.05

^**^p < 0.01

^***^p < 0.001

**Fig. 2:**
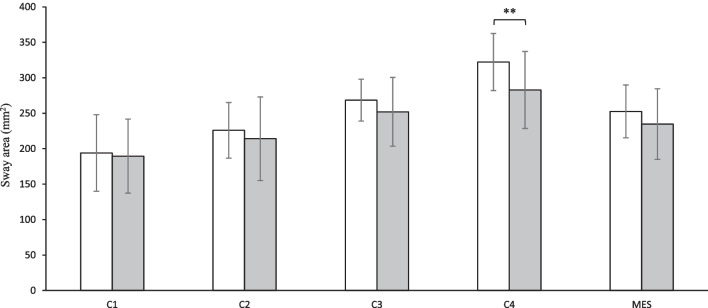
Mean values with standard deviations of postural sway area for four conditions (C1-C4) and mean equilibrium score (MES) in patients (white bars) and healthy controls (gray bars) after MPFL reconstruction; ^**^ *p* < 0.01

The patients’ balance control was restored to almost the same level as that of the healthy controls following the MPFL reconstruction (Fig. 2). A comparable postural sway area between patients (193.9 ± 54.1 mm^2^) and controls (189.5 ± 52.2 mm^2^) was found in C1 (*p* = 0.707, Cohen’s *d* = 0.08). The same finding was also observed in C2 (225.9 ± 39.2 mm^2^ vs. 214.1 ± 58.9 mm^2^; *p* = 0.399, Cohen’s d = 0.24). Patients continued to show similar postural stability (268.5 ± 29.6 mm^2^) when the vision was available to that of controls (252.0 ± 48.5 mm^2^), as the somatosensory was disturbed (C3) (*p* = 0.156, Cohen’s *d* = 0.43). Yet an apparent difference emerged in the subsequent visually unavailable condition (C4) between patients (322.2 ± 40.3 mm^2^) and healthy controls (282.8 ± 54.4 mm^2^) (*p* = 0.005, Cohen’s *d* = 0.82). Overall, no significant differences were observed in MES between patients (252.6 ± 37.3 mm^2^) and healthy controls (234.6 ± 49.8 mm^2^) after MPFL reconstruction (*p* = 0.147, Cohen’s *d* = 0.41). There was no difference in balance control between the two phases of the test in controls (*p* > 0.05).

### Variations in balance control in the study patients before and after MPFL reconstruction

**Fig. 3:**
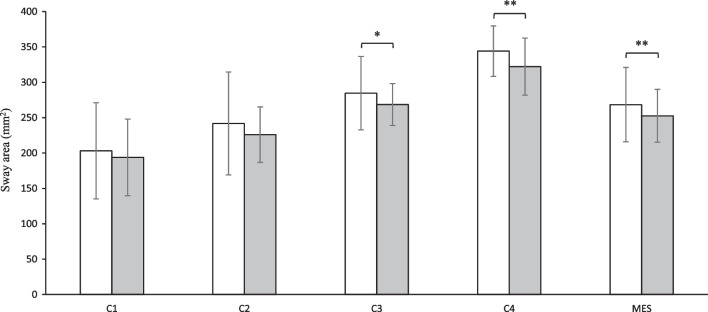
Mean values with standard deviations of postural sway area for four conditions (C1-C4) and mean equilibrium score (MES) in patients before (white bars) and after (gray bars) MPFL reconstruction; ^*^ *p* < 0.05, ^**^ *p* < 0.01

The variations in balance control in the study patients before and after MPFL reconstruction are illustrated in Fig. 3. Significant time effects on postural stability were observed in C3, C4, and MES. Patients displayed comparable sway areas pre- and postoperatively in C1 (203.1 ± 67.9 mm^2^ vs. 193.9 ± 54.1 mm^2^; *F* = 2.333, *p* = 0.139, Cohen’s d = 0.29). In C2, no significant difference in postural stability was observed pre- and postoperatively (241.8 ± 72.8 mm^2^ vs. 225.9 ± 39.2 mm^2^; *F* = 3.045, *p* = 0.093, Cohen’s d = 0.34). As the somatosensory cues were modified, the noticeable divergence of balance control was found with vision availability in C3. Patients had a lower postoperative postural sway (268.5 ± 29.6 mm^2^) in contrast with their preoperative levels (284.6 ± 51.8 mm^2^) (*F* = 5.163, *p* = 0.032, Cohen’s *d* = 0.44). After further loss of visual cues, patients exhibited more significant pre- and postoperative heterogeneity in balance control in this testing condition (C4). A lower postoperative sway area was observed (322.2 ± 40.3 mm^2^) compared to their preoperative level (344.1 ± 35.7 mm^2^) (*F* = 8.213, *p* = 0.008, Cohen’s *d* = 0.56). A lower MES value after MPFL reconstruction (252.6 ± 37.3 mm^2^), in contrast with their preoperative level (268.4 ± 52.5 mm^2^), implied a significantly improved balance control performance as a general reflection of postural stability (*F* = 12.068, *p* = 0.002, Cohen’s *d* = 0.68).

### Clinical assessments of the study patients

**Fig. 4:**
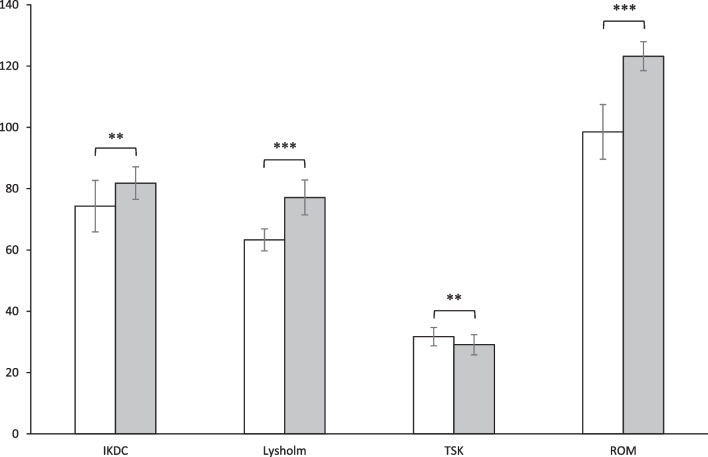
Mean values, associated with standard deviations, of clinical assessments of the affected knee joint observed in patients before (white bars) and after (gray bars) MPFL reconstruction; IKDC: the international knee documentation committee subjective knee form; Lysholm: Lysholm knee scoring scale; TSK: Tampa scale for kinesiophobia; ROM: active range of motion; ^**^ *p* < 0.01, ^***^ *p* < 0.001

In the postoperative follow-ups, all patients had no recurrent dislocations. The outcomes of clinical assessments are illustrated in Fig. 4. Increased IKDC scores in the study patients were observed postoperatively (81.8 ± 5.3) compared to their preoperative values (74.3 ± 8.4) (*p* = 0.002, Cohen’s *d* = 0.95). Likewise, the Lysholm scores also improved significantly after surgery (77.1 ± 5.7 vs. 63.3 ± 3.6; *p* < 0.001, Cohen’s *d* = 2.02). TSK scores were significantly decreased postoperatively (29.1 ± 3.3) in contrast with their preoperative levels (31.7 ± 3.0) (*p* = 0.009, Cohen’s d = 0.57). Meanwhile, active ROM of the affected knee also improved from 98.5 ± 8.9° preoperatively to 123.2 ± 4.7° postoperatively (*p* < 0.001, Cohen’s *d* = 3.13).

Table 4 shows the outcomes of linear regression analysis correlating the pre- and postoperative MES and their clinical assessments. Significant higher correlations were found between balance control and IKDC (*p* = 0.002), TSK (*p* = 0.012), and active ROM scores (*p* < 0.001) following MPFL reconstruction. Furthermore, the preoperative Lysholm score and active ROM also displayed significant associations with postural stability (*p* = 0.007 and 0.023, respectively).

**Table 4:** Linear regression analysis comparing the MES before and after MPFLR along with the clinical assessments

	MES (Pre-MPFLR)		MES (Post-MPFLR)	
	B	*p*	B	*p*
IKDC	− 2.155	0.086	− 4.158	0.002 ^**^
Lysholm	− 7.349	0.007 ^**^	1.715	0.195
TSK	3.839	0.286	5.569	0.012 ^*^
ROM	− 2.628	0.023 ^*^	− 5.747	< 0.001 ^***^

MES: mean equilibrium score; MPFLR: medial patellofemoral ligament reconstruction; IKDC: the international knee documentation committee subjective knee form; Lysholm: Lysholm knee scoring scale; TSK: Tampa scale for kinesiophobia; ROM: active range of motion; Pre-MPFLR: before medial patellofemoral ligament reconstruction; Post-MPFLR: after medial patellofemoral ligament reconstruction; B: unstandardized coefficient

^*^p < 0.05

^**^p < 0.01

^***^p < 0.001

## Discussion

The results of this study were concordant with the hypotheses. Patients with recurrent lateral patellar instability had balance control defects. After MPFL reconstruction, such patients demonstrated a significant improvement in balance control in contrast to their preoperative levels. Moreover, the restored quality of postural stability was comparable to that of healthy individuals.

Conventionally, static balance control in a natural stance requires visual and proprioceptive afferents to provide sensory information [30, 31]. In the present study, patients had balance control deficiencies before MPFL reconstruction compared to the healthy controls, which were primarily concentrated in the testing environment where proprioception was disturbed. The injured knee joint was unable to effectively perceive the proprioceptive cues when the study patients stood on a foam support surface, resulting in a lack of rapid response and coping strategies to adjust body equilibrium, thus, leading to poor balance control performance. However, patients exhibited normal postural stability, regardless of whether the vision was available or not, when standing on a firm support surface. This suggests that visual cues provide a limited reference for static balance control, with the human body primarily relying on the processing of somatosensory inputs by the central nervous system (CNS) to regulate standing balance. This process plays a dominant role in postural regulation, consistent with the conclusions reported in the literatures [32–34]. Almost no difference in balance control between the study patients and healthy controls was observed when the MPFL reconstruction was performed. It indicates that the semitendinosus autograft can reconstruct and replace the original MPFL’s proprioceptive conduction and re-participate in the postoperative balance regulation, producing the same regulatory effect as that of healthy individuals.

A comparison of pre- and postoperative balance control in the study patients also reflected the heterogeneity of postural stability in a somatosensory-disturbed environment. When the proprioceptive cues were available, the residual MPFL could also effectively sense the proprioceptive inputs and participate in the balance regulation, and the effect of this regulation was comparable postoperatively to that of the new autograft. Once the somatosensorial afference was interfered with, the injured MPFL was unable to appropriately perform postural control, while the reconstructed new ligament can effectively perceive the proprioceptive cues and produce a new compensatory effect on balance control. In contrast, increased postoperative IKDC and Lysholm scores suggest improvements in symptoms, joint efficiency, and motor ability after restoring ligament morphology and structure, from a clinical and functional point of view. Furthermore, the complete ligament structure and firm fixation ensure significantly reduced apprehension for recurrent dislocation of the patella and greater active ROM after surgery; thus, MPFL reconstruction can improve patients’ balance control in addition to ameliorating the proprioception of the knee joint. The subsequent linear regression analysis statistically proved that the improvement of joint function in patients was closely related to their postural stability, and this improvement attributed to the new grafts would ensure the patients regained satisfactory postoperative balance control.

The pathogenesis of instability of the patella is complex and multifactorial [26, 35]. A delicate balance of a series of structures involving bone and soft tissue ensures the patellofemoral joint stability, and upsetting this balance can result in the emergence of a pathological state. As the understanding of the MPFL and its role in guaranteeing the mediolateral stability of the patella continues to progress, surgical reconstruction of the MPFL provides patients with a feasible and promising alternative [12, 36]. To avoid the impact of bony interventions such as tibial tubercle osteotomy, trochleoplasty, or derotation osteotomy on balance control in patients with patellar instability, patients with no significant anatomical abnormalities were only selected as the study participants, aiming to investigate the role of the autologous tendon graft in balance regulation. In the choice of graft, in addition to our surgical experience in anterior cruciate ligament (ACL) reconstruction, the preference for the semitendinosus tendon in MPFL reconstruction is also owing to its anatomical and biomechanical advantages. The semitendinosus provides a longer tendinous portion and more resistance to the traction, with reduced elastic modulus compared to both MPFL and gracilis tendon; therefore, elucidating the reduced tendency to redislocations and revisions [37, 38]. In addition, the quadriceps tendon was not selected as the graft, contrary to the literature, to avoid postoperative anterior knee pain and reduced extension strength, at the cost of failing to preserve the patellar bone insertion and adding a new harvesting site incision [39].

A safe return to sports following MPFL reconstruction is unclear, but it does improve the ability to carry out daily tasks as usual [40]. The rehabilitation criteria of ACL reconstruction are typically cited by surgeons and physical therapists when making decisions [19, 41]. A full range of motion, no postoperative recurrent patellar instability, a good neuromuscular response, good balance control, and exceptional lower limb strength are just a few examples of the common cognition involved. Similar to the current study, Lion et al. found that 19 ACL reconstruction patients, as opposed to 21 healthy controls, were able to regain normal bipedal static balance control 9.2 months after surgery [28]. However, one year after autogenous osteochondral mosaicplasty, patients with cartilage defects of the knee in our prior study were unable to regain their pre-injury level of balance control [29]. The cartilage injury in the weight-bearing region of the knee, which is distinct from MPFL or ACL injuries in the non-weight-bearing region, may be the cause for the discrepancy in the results. This injury causes deviations in the proprioceptive and compressive stress signal perception by the mechanoreceptors in the osteochondral structure. After cartilage transplantation, morphological healing takes place; however, the signaling along the entire sensorimotor chain is still lacking, preventing the normal restoration of balance control. Through the use of mechanoreceptors like Pacinian corpuscles, Ruffini endings, and Golgi organs, the ACL has a somatosensory function that communicates proprioceptive information about joint position to the CNS [42]. However, there is no conclusive histological proof that the MPFL and semitendinosus tendon contain mechanoreceptors. So, a theory was developed to explain the cause of patellar instability [43]. This theory postulated that when MPFL is damaged, the Golgi organ or muscle spindle in the knee cannot effectively sense the lengthening or shortening of the patella tendon, the quadriceps femoris muscle, or the joint capsule. As a result, the ipsilateral cerebellum’s activity is decreased, and the ascending afferent pathway through the spinocerebellar tract, one of the somatosensory pathways, is also reduced. Based on this theory, we speculate that the transplanted semitendinosus tendon acts as a new MPFL replacer, reestablishing signaling in the sensorimotor chain of the knee and contributing to the restoration of postoperative balance control.

There were some limitations to this study. In patients with recurrent patellar instability, we only looked at the effect of MPFL reconstruction on static balance control; we did not pay attention to the patients’ dynamic postural stability, which is crucial for their postoperative daily life. More balance control tests were not performed postoperatively due to the lack of a standard timeline for graft healing and returning to sports, making it impossible to observe the variation in their postoperative postural stability. In addition, single-leg stance balance tests were not performed in the present study, which may be a better way to check how the affected limb was behaving and compare it to the unaffected extremity. Future research should focus more on the diversity of balance control tests and its dynamic changes to enable more logical and scientific patient management.

## Conclusions

In conclusion, the study findings demonstrated inefficient balance control in patients with recurrent lateral patellar instability as compared to healthy individuals. The static bipedal balance control is improved under surface perturbation in these patients one year after isolated MPFL reconstruction, and the possibility of normal restoration of postural stability is enhanced. The ligament’s structural recovery could help restore the sensorimotor efficiency and generate compensatory and anticipatory balance regulation strategies. Thus, the knee can restore its morphological and functional stability, assisting surgeons and physiotherapists in making disease management decisions and patients’ return to sports and daily life.

## Data Availability

All data generated and analyzed during this study are available from the corresponding author on reasonable request.

## Abbreviations

MPFL: Medial patellofemoral ligament
IKDC: International Knee Documentation Committee
TSK: Tampa scale for kinesiophobia
ROM: Range of motion
CT: Computed tomography
Q angle: Quadriceps angle
CoP: Center of foot pressure
MES: Mean equilibrium score
CNS: Central nervous system
ACL: Anterior cruciate ligament
